# The potential of small-scale spatial data in regional science

**DOI:** 10.1007/s10037-022-00172-3

**Published:** 2022-10-17

**Authors:** Rolf Bergs, Rüdiger Budde

**Affiliations:** 1 Policy Research & Consultancy, Im Hopfengarten 19 B, 65812 Bad Soden, Deutschland; 2 RWI – Leibniz Institute for Economic Research: RWI – Leibniz-Institut für Wirtschaftsforschung e.V., Hohenzollernstraße 1–3, 45128 Essen, Deutschland

Because of the growing complexity in the various dimensions of social life, policy is increasingly challenged with designing prudent solutions for the economy, the environment, public health, education, housing, and further fields of intervention. This issue applies to all spatial scales (local, regional, national). Policies need evidence-based, reliable and relevant information (Leopoldina 2019). Sufficient information is directly related to the available data. The granulation and geographical scale at which data are available are both of overarching importance. It was thus worth launching a special call for papers to contribute to the ongoing debate and to add fresh knowledge about selected topics of the subject. The call deliberately addressed a broader scope of relevant issues as we have been aware that a complete mapping of all facets of data types and appropriate empirical methods would certainly go beyond a small project such as a special journal issue. To fill this gap, we have decided to write an extended editorial that covers all facets of the call’s content in an introductory depth. The four methodologically and technically relevant manuscripts present research results that deepen individual topics of the call with specific research questions and methodology.

Until recent times a major constraint in spatial analysis has still been the information deficit at the small-scale spatial level. Due to insufficient evidence, prudent policies were constrained at both local and global levels. Socio-economic data at the administrative regional or district level have been mostly too coarse to differentiate the situation in areas below the level of districts. At the municipality or neighborhood level, the range of data—if available at all—has been too narrow and just relevant for descriptive statistics, such as population change and density. At the level of the spatial aggregate (region, nation), data is broadly differentiated along with a large number of variables observed; however, the information content of those data is likewise reduced because of unobserved major or minor errors around the observable averages and sums. Targeted interventions to support the economy, social security, the labour market, or to stave-off detrimental effects on the environment or public health are thus essentially imprecise.

All this has triggered debates in many developed countries about how best to improve data systems. The aim of official data is to report on the frequency of certain characteristics related to a point, a time period, or a spatial dimension. Often, major effort is spent on elaborating and recording the content-related criteria, while administrative boundaries represent the parameters of the spatial dimension. In empirical research carried out with such statistics, it is assumed that the choice of the reporting area does not influence the characteristics observed. However, it turns out that there are often interdependencies between the frequencies of the explored characteristics and the choice of spatial reporting units, which often significantly impact empirical results.

Therefore, spatial planners and regional economists try to derive their diagnostic units from the existing small-scale statistics to make sure that they can analyse and evaluate their investigation facts within the structures created in this way to be able to derive findings, options for action, and needs for action (Karl and Klemmer 1990, p. 36). The aim is to divide larger spatial units into smaller spatial units of analysis to identify spatial characteristics or interdependencies.

Since aggregations are already made in the preparation of the basic statistical material, potentially important information can get lost in the data generating process, such that this will definitively affect the subsequent empirical analysis. In addition to this “traditional” data, novel geodata opens up promising research opportunities in social and economic science research. Although data of this type, like the previously discussed data, has a content-related, temporal, and spatial dimension, the spatial reference is precisely mapped via geographical reference systems (coordinates) and object type. Through these location parameters, it is possible to increase the explanatory value significantly. Besides the improvement of spatial data, rapid progress in information technology (such as geo-informatics and artificial intelligence), theory (such as new economic geography modeling), and empirical methodology (such as statistical classification tools and advanced spatial econometrics) have opened up new avenues towards better spatial information.

Due to those technological and methodological advances, a growing number of researchers use the opportunity to incorporate geospatial data into spatial research using geographic information systems (GIS). As a result, the database of small-scale statistics has been expanded to include topological landscape models, data from national surveys, and earth observation using satellite data. Since the beginning of this century, there has been a growing availability of small-scale data at the neighbourhood level and raster data with a resolution of one or less than one square kilometer. All this has led to addressing research questions with much higher levels of precision.

Public authorities have already acknowledged the advantages of higher resolved spatial data for policy. Meanwhile, Eurostat and all national statistical agencies in member countries grant free access to small-scale grid data on population density. Additional data with the risk of individual recognition and de-anonymization are usually restricted to scientific and administrative use only (just as for micro-data in general). However, among countries, different strict data protection regulations have also led to a different depth and scope of data portfolios for variables other than just demographic ones. In Nordic countries, further differentiated socio-economic data at the grid or small-scale level are freely available, such as on enterprise locations in Finland. Here spatial data on enterprises are offered in accordance with the INSPIRE data specification on production and industrial facilities (2-digit Standard Industrial Classification level). In Germany, commercial small-scale databases with specific socio-economic variables are available (e.g., the datasets from Microm Consumer Marketing); meanwhile several such datasets have been purchased by research institutes and statistical authorities to complement research data and official statistics. Moreover, there is ubiquitous free access to various sorts of day and night satellite imagery provided by authorities in the US, Europe, China, and other countries. Worth mentioning is highly precise global night satellite imagery with a resolution of a few hectares per pixel. This Special Issue also addresses this type of data (see below).

Two major questions are at the center of this Special Issue: (i) How is small-scale spatial data generated, and which are the appropriate specific statistical and econometric methods? And (ii) What are the dimensions of benefit where the availability of small-scale spatial data can contribute to better policies?

## Data types, generation of small-scale spatial data, and the range of empirical methodologies

In today’s research, the data supply of the official statistical offices forms the core basis for spatial analysis. In particular, the large-scale censuses provide the opportunity to put other data collections into an overall context. Census and survey-based data are the first important family of small-scale spatial data. Several developments that evolved independently have promoted the improvement of the supply of such traditional small-scale spatial data.

1. Many public authorities worldwide provide some of their stored geographic data (often compiled over decades) on an open-access basis.
2. On the internet, an increasing flood of information is provided. By using methods like web scraping (Gunawan et al. 2019), the number of possible data sources has risen to such an extent that it becomes indispensable to call their quality into question.
3. Further data sources can be explored for scientific research by conducting targeted field research through structured interviews.
4. Other kinds of information can be obtained by analyzing flows of goods from mail-order businesses and publishers or queries to search engines. Data traders use these sources since they provide essential information with strategic value at the firm level and for local policies.
5. New data sources can be generated by transferring them to a geographical system using the stored addresses. By using such methods (also known as “geocoding” or “georeferencing”) (Goldberg 2008), it is possible to enrich existing spatial information with further attributes. Since this technique is relatively simple, it is now possible to tap into a broad spectrum of additional data sources.

By using all these means, it is possible to delineate study areas so that the spatial units contribute to uncovering the specific phenomena under study.

Small area level estimation is vital in light of a continual demand by data users for exact geographic detail in the published statistics and for various subpopulations. Traditional demographic sample surveys designed for national estimates do not provide large enough details to produce reliable direct estimates for small areas such as counties and even larger regions. Advanced statistical models can generate small area estimates with greater precision; however, the bias due to an incorrect model specification or failure to account for informative sampling needs to be considered.

In fact, a great deal of such kind of small-scale data is produced by virtue of statistical estimation. Here advanced estimation techniques have been introduced and further refined during the last ten years, such as e.g., entropy econometrics (Fernández-Vázquez and Rubiera Morillon 2012; Zhang et al. 2014) or—yet more recently—data imputation by machine learning to precisely estimate gaps in data or generate granulated artificial data that comes significantly close to real data. Generative Adversarial Networks (GAN) are a promising tool (Klemmer et al. 2019).

A second family of small-scale spatial data is the realm of sensor-generated data. These are produced by ground-based technologies (e.g., air pollution, gauge, or rain observation stations) or remote sensing such as aerial images or satellite imagery. Day and night satellite data provided by the Sentinel missions (Copernicus) or the NOAA^1^ are well-known examples. Worth mentioning are, e.g., daily global images of greenhouse gas emissions (Sentinel 5P), the DMSP-OLS composites provided by the NOAA until 2011/2012, and the new 14-bit on-board calibrated VIIRS images with a much stronger precision. The high spatial correlation between light emission and several socio-economic and environmental variables is a major advantage of using those data in socio-economic studies. Often, a direct environmental relationship is assumed between light emission and light pollution, the latter being associated with hive death or the disappearance of other insect species. Intuitively, nocturnal luminosity is also closely associated with anthropogenic activity, like the level of production, service provision and distribution by transport infrastructure (i.e., economic activity), energy consumption, and the related level of greenhouse emissions (Henderson et al. 2011; Chen and Nordhaus 2011; Mellander et al. 2015). Pinkovskiy (2017) used DMSP-OLS composites to estimate the structural breaks of economic growth along borders by applying a regression discontinuity design on light emission. The images and estimates presented in this study impressively show the diverging growth trends along the Ukrainian borders to the EU candidates Poland, Hungary, and Romania during 1992 to 2000. Proville et al. (2016) explored various relationships between light emission and economic activity by correlation analysis. As expected, electricity consumption and CO_2_ emissions are correlated most strongly with light emission (0.93). However, the correlation with GDP is only slightly lower (0.91). At first glance, such high levels of association would suggest using such digital imagery to estimate missing socio-economic data at a higher resolved scale. A major constraint for establishing stable relationships between different sorts of spatial data is spatial heterogeneity, such as varying land use or population density. All this implies a varying strength of correlation between luminosity and socio-economic and environmental variables along with different classes of land use. Machine learning technologies may open doors for an improved understanding of spatial data and its intrinsic relationships at different spatial scales.

Nevertheless, in developing countries with poor official data infrastructure, DMSP and VIIRS have already played an important role in estimating income or environmental pollution (Ghosh et al. 2013). This Special Issue includes two papers using night satellite images, one is a study on Namibia exploring night light as a proxy to estimate wealth at a small-scale spatial level (Cf. the paper of Määttä et al. in this Special Issue).

While survey and census-generated data have always been subject to anthropogenic error risk, such sensor-based data are likewise affected by possible errors. For satellite images, auroral light with disturbing artifacts, technical malfunctions of the sensor, pixel value outliers, light overglow, implications from different sensor technologies, and changing lighting technology are worth mentioning. Error removal in night sat images is possible (such as inter-calibration of image time series with improving sensor quality, removal of blooming, or removal of outliers) but can also be quite challenging, such as a correction of distorted variance implied by different lighting technologies, such as traditional lights versus LED (Kyba et al. 2017).

The projection selected is also an essential criterion for properly interpreting the data. Pixel size adjustment is especially important for the observation of regions with a large latitude range because actual pixel sizes on the ground decline with growing distance from the equator. Hence, sensor-generated data should never be used under the prior assumption of “ready-to-use” and flawlessness.

A third family of small-scale spatial data is hybrid data. Here survey and sensor-generated data are merged or further processed. Many such databases are open-access and presented by online viewers. Well-known examples are the EU CORINE database on land use. At Columbia University, the Socioeconomic Data and Applications Center (SEDAC) offers a broad range of global small-scale spatial data. One recent complement has been the NASA Covid-19 viewer (https://sedac.ciesin.columbia.edu/mapping/popest/covid-19/). This database provides not only actual data on the spatial epidemiology of Covid-19, but also additional data on particulate matter at the endpoint, and 2020 population density at any scale and geographical clipping. Even though the scale of data differs among countries, the different data layers offer important insight into relationships between the local environment, population, and the pandemic. Further new composite databases comprise the Global High Resolution Daily Extreme Urban Heat Exposure. Combined with similarly resolved population data, this database offers a powerful tool for monitoring climate hazards and local resilience (https://sedac.ciesin.columbia.edu/data/set/sdei-high-res-daily-uhe-1983-2016).

The IÖR-Monitor, run by the German Leibniz-Institute for Ecological Urban and Regional Development, offers valuable small-scale spatial data on local land use, the density of road and rail networks, sustainability contexts, disaster risk, and renewable energy (https://www.ioer-monitor.de/). So far, the monitor results of this research are only presented for Germany, but they could potentially be provided at a larger geographical scope.

In addition, there have been research projects funded by the European Commission exploring the use of satellite data and merging those with other microdata available. Such hybrid databases have been especially used for evaluation and controlling in rural and agricultural development, (Brennan et al. 2016; Bozzo et al. 2019; OECD 2019; Finger and El-Benni 2021; Michalek 2022).

The above-structured features of small-scale spatial data are only a spotlight on selected sources; The discussion of the entire spectrum of accessible spatial databases would go far beyond the scope of that Special Issue. The main advantage of such small-scale spatial data is that they allow exploring evidence of different properties of space, namely spatial heterogeneity and spatial dependence, in a considerably higher level of detail. Spatial heterogeneity has a much broader scope than presented in the few articles in this Special Issue. Landscape, topography, climate, the population distribution, to mention some, determine the heterogeneity of space. One important field of research in the context of the distribution of population is functional space, especially the distribution of urban and non-urban space. A broad spectrum of analytical tools has been developed for this sub-area of research to process small-scale spatial data and make truly functional space visible.

Similarly, the other property of space, spatial dependence, can be well explored by geostatistical tools using granulated spatial data. Standard methods comprise the comparison of local and global spatial autocorrelation of different variables and spatial econometric modelling of highly resolved data (Dubé and Legros 2014). Spatial econometric modeling has dynamically evolved during the last decades, going beyond the standard models that just view the right or left side of the equation (such as Spatial Error, Spatial Autoregressive, or Spatial Lag of X models). The different types of models simultaneously viewing spatial dependence of the dependent variable, the predictors, and the residuals allow us to choose among tailor-made procedures for the respective purpose.

## The different dimensions of the use of small-scale spatial data

### Segmentation and classification of space

In recent years there have been lively research activities to solve the mismatch between static administrative and dynamic natural space, especially for detecting true urban areas. While in former times, the primary parameter of urban-rural delineation was the common administrative boundary between both types of land use, it is now possible to observe dynamic urbanization processes by spatial segmentation and classification techniques. Meanwhile, quite a broad spectrum of different methodologies has evolved. Unsupervised classification comprises traditional cluster analysis like k‑means or Isodata, e.g., to segment urban and non-urban space based on population density data or night satellite images, classify land use, or detect pollution-affected regions (e.g., Small et al. 2006; Bergs 2018). Furthermore, CLARA (Clustering for Large Applications) and kernel density estimation are methods to classify space (e.g., Budde 2018; Budde and Neumann 2019). Several other statistical methods have been proposed.

Supervised classification builds on known patterns on which software can be trained to detect classes in images or databases. The dominant approach is to set the parameters of a model such that its likelihood is maximized (maximum likelihood estimation). Supervised and unsupervised machine learning techniques to analyze geographical space and its change have enormously advanced in recent years with a dynamically growing number of international conferences dealing with GeoAI (Lavallin and Downs 2021). Worth mentioning are *inter alia* techniques such as minimum spanning trees, random forests, k‑nearest neighbours, artificial neural networks, and support vector machines (e.g. Assunçao et al. 2006; Sun et al. 2020; Rybnikova et al. 2021; or for an overview: Kopczewska 2021). Meanwhile, GIS, image analysis, and advanced statistics software comprise such geostatistical classification tools. The Zotero database provides a large fund of respective literature, e.g., for studies using satellite imagery (https://www.zotero.org/groups/2913367/alan_db/library). This Special Issue includes a paper with a further novel classification methodology, namely topologically connected space applied to nocturnal satellite imagery (cf. the paper of Spinosa in this Special Issue).

An important innovation and benefit of such data analysis techniques for policy is the enormous gain in information. Trends of urban sprawl, threats like environmental damage due to excessive soil sealing, or land use conflict along the urban fringe can be timely addressed by better informed local and regional policies to prevent unfavorable spatial trajectories.

### Reconciling spatial heterogeneity and dependence

Traditionally, spatial heterogeneity has been defined as the first-order property of a spatial process moment of space (the deterministic dimension), while spatial dependence constitutes its second-order moment (the stochastic dimension). Small-scale spatial data is capable of better revealing central issues of both deterministic as well as stochastic properties of space. In a very recent paper, Furková (2021) shows how spatial innovation spillovers determine the spatial distribution of patents. Furthermore, space is heterogeneous in a different way, not just simply by an immediately observable diversified landscape, by innovation levels, variation of climate or relief, etc., but also by a peculiar hidden spatial heterogeneity. In other words: While the distribution of cities on a map appears chaotic to the observer, the underlying rank-size distribution of them is surprisingly uniform in any country and constitutes a scaling law (Zipf’s law) that has been the subject of lively research in regional science and urban economics since the 1950s. It represents a quasi-natural law still regarded as an enthrallment of rare social physics. Considering this quasi-natural phenomenon has opened avenues in research to reconcile scaling law in space and spatial dependence (Jiang 2017). Or as Zhang et al. (2014) point out: “… It will be seen that the way forward lies in the integration of geo-statistics and process model-based methods in which spatial dependence and spatial heterogeneity are characterized and incorporated in the description and modeling concerning the underlying processes that control the information dynamics …”. From the viewpoint of policy and planning, a holistic application of spatial heterogeneity and dependence improves the information on the dynamic of spatial evolution., such as, e.g., integration versus disintegration processes at borders or urban sprawl.

From the viewpoint of analytic methodology, advanced spatial econometrics will play a significant role at the intersection of spatial heterogeneity and spatial dependence. The paper by Neumann and Taruttis (in this Special Issue) applies a spatial autoregressive model with autoregressive disturbances (SARAR) to explore hedonic pricing in urban neighborhoods^2^. Spatial dependence of model variables and residuals (represented by the range of spatial econometric models) provide more insight into determinants of particular spatial heterogeneity, such as scaling laws of cities (Rybski and Gonzalez 2021; Arcaute and Ramasco 2021). A further pertinent econometric tool to process small-scale spatial data is the Geographically Weighted Regression (GWR), both in its basic and multiscale implementation (cf. the paper of Ho and Wilhelmsson in this Special Issue). GWR can also be directly connected to spatial econometric models (Furková 2021).

### Spatial interaction

Spatial interaction is largely determined by function. There is an essential reason why cities and rural areas are distributed in space as they are: they exhibit functional synergies. The evolving distribution of urban and non-urban space was already described by Zipf in his seminal contribution on the rule of least effort (Zipf 1949). Rural-urban interaction is primarily determined by nature and the effort needed to bridge distances between cities. In simple words, without cities, there would be no rural areas, space would be homogenous, and people would not move. More recent models of geographical economics show that the core difference between living in a city and living in rural areas constitutes fewer transport efforts in cities, scale economies, and the “love for variety” effect. Apart from food consumption, urban dwellers can choose from a larger variety of substitutable manufactured goods; and this defines an urban amenity.

However, there is a point of equilibrium in the distribution of urban and rural populations. Otherwise, all people would settle in cities. Here, the functional differentiation and the corresponding interaction between places become central. With a particular focus on rural-urban synergies, a recent EU-funded research project could generate some corresponding insight. In fact, highly resolved spatial data allows the direct observation of functional networking and spatial interaction. Budde (2018) shows for the Rhein-Main region how commuter flows present themselves among different statistically classified spatial categories in the urban—peri-urban—rural continuum. Issa and Bergs (2022) use Sentinel 5P image composites showing NO_2_ emissions (14 days moving averages of daily images from March to April 2020), local data on Covid-19 incidence, and the local level of teleworkability to estimate the potential reduction of greenhouse gas emissions in German regions exhibiting different economic structures. Using municipality-level data of de-population risk linked to a survey in the Valencia region (Spain) during the Covid-19 period, Ruiz-Martinez and Esparcia (2020) show how functions and economic prospects of rural regions depend on the local internet access.

### Cross-sectional neighborhood effects

The topic of neighborhood effects has been addressed in the literature for over 25 years. Economic development in a region is not only seen as a function of its endowment of potential factors. It also influences the evolution and endowments in neighboring areas. For example, Ellen and Turner (1997), Galster (2002), Dietz (2002), Durlauf (2004), and van Ham et al. (2012) have already examined social or health economic phenomena such as educational success, school dropout rates, deviant behavior, social exclusion and the like with different approaches across regions.

Researchers have to face the task of identifying actual causal effects when studying neighborhood phenomena (Durlauf 2004). This task shifts the scientific focus from findings from studies that use qualitative methods to those that use quantitative techniques. Because of these different approaches, qualitative research produces exemplary results, often leading to ‘best practice’ recommendations, while quantitative research aims to identify independent, generalizable causal mechanisms (Moffitt 2001; Durlauf 2004). In particular, the informative value of quantitative research results depends on how much it solves the selection problems at hand: On the one hand, the choice of variables used in the research project may constitute a challenge; they should measure the phenomena to be investigated as precisely as possible. Depending on the research approach, different criteria are used to map the complex characteristics of the phenomenon of neighborhoods: Manski (1995) divides them into “endogenous”, “exogenous”, and “correlated” categories. Ellen and Turner (1997) divide them into five categories: Concentration, location, locational, physical, and services. Leventhal and Brooks-Gunn (2004) use the rubrics “institutional resources”, “relationships”, and “norms/collective efficiency”. According to Galster (2012), the descriptive variables should cover social interactive, environmental, geographical, and institutional categories.

On the other hand, the design of the econometric model world has a decisive influence on the statistical quality of the results. The more it succeeds in reflecting reality in the modeling, the more meaningful the results (see Elhorst 2014; Anselin and Williams 2001).

### Spatial epidemiology

Prompted by Covid 19, spatial epidemiology has received growing attention. Since mid-2020, the European Regional Science Association (ERSA) has been collecting respective research on its website to contribute to combating Covid-19 from the part of regional science (https://ersa.org/forum-coronavirus/). Furthermore, ERSA has launched a “Covid-19 Battle Series” of lectures. Small-scale spatial data have played a particular role in Covid-19 spatial epidemiology and studies viewing the impacts of Covid-19 on the economy, the environment, or other spheres of life. Mobility has constituted an important predictor of the rate of new infections. The more mobility, the more contagion can be observed. A useful predictor database has been the changing spatial dynamic of mobile phones detected (e.g. https://www.covid-19-mobility.org/mobility-monitor/). It is to be stressed that even in the context of the pandemic, the concept of a functional area has proved to be highly relevant for policies to combat Covid-19. Iacus et al. (2021) have shown for Austria that highly interconnected zones, representing a mobility-determined functional area, exhibit a significantly higher infection incidence than the rest of the country. Apart from that, the regional sectoral structure of the economy, the magnitude of local air pollution, local cultural habits, or the dominant local political leanings appear pretty often as further significant predictors of the local infection incidence. Apart from Covid-19, highly resolved spatial data on the prevalence of chronic diseases and data on the local environmental burden, such as pollution, noise, and light emission (even differentiated building types), may provide evidence for policy to improve public health protection.

### Policy impact estimated with small-scale spatial data

Classical tools of policy impact analysis relate to the range of different causal inference techniques such as propensity score matching, difference-in-difference regression, regression discontinuity design, or estimations with instrumental variables. In those models, spatial dependence and heterogeneity influences are neglected largely and may occasionally induce the risk of violating the stable unit treatment value assumption (SUTVA). Kolak and Anselin (2020) have shown the important spatial relevance of policy impact with the illustrative example of minimum legal drinking age and mortality. To our knowledge, there has been little research yet using small-scale spatial data in spatially extended causal inference estimation procedures.

When using small-scale spatial data for empirical research, local policy intervention can also be a variable that could enter a spatial econometric model as a continuous, categorial, or binary predictor. If the intervention’s point of time and place (geographical coordinates) are provided in micro-data sets, the net effects of policy can be further corrected by controlling for spatial autocorrelation in standard estimation procedures. This way, a potentially powerful tool of local and global policy scenario analysis could emerge in addition to the standard application of various causal inference analysis procedures applied in policy evaluation.

To illustrate the high potential of small-scale spatial data for spatial economic analysis and policy, we have invited specialists to submit specific studies for this Special Issue of the Review of Regional Research.

## The four papers of this Special Issue

The range of themes defined beforehand is too broad to be covered in this Special Issue. Nevertheless, it was possible to highlight three kinds of central purposes of small-scale spatial data: classification of space, spatial heterogeneity and dependence, and cross-sectional local effects. The four papers in this Special Issue thus cover selected facets of the potential of this data. Novel data types and methodology are particular foci of all four papers.

The paper “Sorting in an urban housing market—is there a response to demographic change?” determines changes in the price levels of residential rents and house purchases at the district level of the city of Dortmund. As a data basis, the condition and development of the properties on offer as well as the size and development of the population are collected at the grid level of one square kilometer. The property data are converted to 5‑digit postal districts, while the demographic data are only available at the level of postal districts. This small-scale data makes it possible to estimate supply prices at the district level by hedonic calculations. At the same time, the discrete choice model is used to determine the probability of whether and under what conditions a specific household type will settle in a particular area. The approach presented by Uwe Neumann and Lisa Taruttis enables them to identify the effects of aging and mortality and environmental measures.

The general idea of what is meant by an urban zone is quite broad. It ranges from administrative units to homogenous districts or functional areas defined according to specific characteristics. However, the prevailing assumption is that a historically developed administrative delimitation is too narrow for today’s analytical questions and that “wider urban zones” should come into focus. This raises the academic question of which delimitation method is the most appropriate.

In the paper “Wider urban zones: use of topology and nighttime satellite images for delimiting urban areas” Andrea Spinosa uses nighttime satellite images to define urban areas as coherent topological spaces. He proposes a universally valid iterative algorithm that leads to comparable spatial definitions, regardless of which states or spaces are being compared. The approach should make spatial comparisons possible without any regional bias.

The paper on “Nighttime lights and wealth in very small areas: Namibian complete census versus DHS data” by Määttä, Ferreira and Lessmann addresses Namibia as a particular developing country. The findings reveal the substantial utility of sensor-generated data as compared with the traditional survey-generated statistics. The authors show that light emission at a highly resolved spatial level correlates much stronger with complete census data than with the sampled Demographic and Health Survey (DHS). So far, the association between wealth and light emission has been demonstrated at the level of larger aggregates (notably at the national scale). The findings of this new paper prove night light data to be a quite reliable tool to estimate wealth even at the local level in a developing country.

In their paper on “Geographical accessibility to bank branches and its relationship to new firm formation in Sweden via multiscale geographically weighted regression”, Ho and Wilhelmsson demonstrate the methodological power of multiscale geographically weighted regression (MGWR) in processing small-scale spatial data. Local banks and the related etablishment of new enterprises are at the center of the study. Based on Swedish municipal data describing numerous variables such as new firm formations (dependent variable), distance to bank branches, income, employees, education level, industrial specialization, immigrants etc., the authors show the geographical distance to the nearest bank branch to be negatively associated with firm formation. To avoid the apparent problem of reverse causality and thus endogeneity of the variable “accessibility to nearest bank branches”, the authors employ an instrumental variable approach augmenting their MGWR model.

We hope that this collection of papers appeals to scholars and practitioners from urban economics, economic geography, regional political economy and other prongs of regional science to engage in the debate about better spatial data and contribute to the generation of further fresh knowledge.

## Footnotes

1 The DMSP-OLS (Defence Meteorological Satellite Program—Operational Linescan System) and VIIRS (Visible Infrared Imaging Radiometer Suite) composites were originally administered by the NOAA (National Oceanic and Atmospheric Administration). For research purposes those databases and special derivates are in the custody of the Payne Institute at the Colorado School of Mines. Supranational organizations like the European Commission monitor land use within Europe via satellite images. The reporting grids vary from several square meters up to just one square kilometer. Some organisations of the aerospace sector share a range of products on open access basis, such as imagery of the Sentinel missions of the European Space Agency (ESA) provided as raw data on a daily base or displayed as moving averages.

2 Meanwhile spatial econometric theory modelling has advanced further, now arriving at dynamic general spatial panel nesting models with principal component determined common factors (Elhorst 2022). Even though extremely complex and challenging, such models might open doors for most precise estimates with multi-dimensional dependencies at smallest spatial scale.
